# Association of Gestational Opioid Exposure and Risk of Major and Minor Congenital Malformations

**DOI:** 10.1001/jamanetworkopen.2021.5708

**Published:** 2021-04-13

**Authors:** Xuerong Wen, Nicholas Belviso, Emily Murray, Adam K. Lewkowitz, Kristina E. Ward, Kimford J. Meador

**Affiliations:** 1 Health Outcomes, Department of Pharmacy Practice, College of Pharmacy, University of Rhode Island, Kingston; 2 Maternal Fetal Medicine, Women and Infants Hospital of Rhode Island, Alpert Medical School of Brown University, Providence; 3 Department of Pharmacy Practice, College of Pharmacy, University of Rhode Island, Kingston; 4 Department of Neurology, Stanford University, Palo Alto, California

## Abstract

**Importance:**

The rapid increase of opioid-related overdoses and deaths has become a public health concern in the US. Use of prescription opioids in pregnant women has increased; results from teratogenicity studies remain controversial.

**Objective:**

To evaluate the association between maternal prescription opioid use (excluding opioid use disorders) during pregnancy and the incidence of congenital malformations.

**Design, Setting, and Participants:**

This retrospective population-based cohort study evaluated linked Rhode Island Medicaid claims and vital statistics data of live births from January 1, 2008, to December 31, 2016. Data analysis was conducted from May 1, 2019, to May 31, 2020. Women who had a live birth during the study period, but no cancer or opioid use disorder, were followed up from 3 months before pregnancy to the end of pregnancy.

**Exposures:**

Data on the mother’s prescription opioid exposure were obtained through pharmacy claims and exposure was defined as dispensing of at least 1 prescription opioid during the first, second, or third trimester.

**Main Outcomes and Measures:**

The primary outcome was overall major or minor congenital malformations, defined as 1 or more major or minor congenital malformation. Secondary outcomes were defined as 10 specific categories of congenital malformations classified by organ systems using *International Classification of Diseases* diagnosis codes.

**Results:**

Of 12 424 included pregnancies, 891 mothers (7.2%) received prescription opioids during pregnancy and 3153 infants (25.4%) were diagnosed with major or minor congenital malformations. Comparing prescription opioid exposure vs nonexposure, no excess risk was observed for major birth defects in infants with opioid exposure in trimester 1 (adjusted relative risk [aRR], 1.40; 95% CI, 0.84-2.34), and higher risks were found for overall minor birth defects in trimester 3 (aRR, 1.26; 95% CI, 1.04-1.53) and minor birth defects in the musculoskeletal system in trimester 2 (aRR, 1.50; 95% CI, 1.10-2.03) and trimester 3 (aRR, 1.65; 95% CI, 1.23-2.22). Significant dose responses in selected minor malformations and effects of specific opioids were also identified. Hydrocodone in trimester 2 (aRR, 3.01; 95% CI, 1.80-5.03) and oxycodone in trimester 3 (aRR, 2.43; 95% CI, 1.37-4.02) were associated with plagiocephaly, polydactyly, and other specified congenital deformities of the hip.

**Conclusions and Relevance:**

The findings of this study suggest a higher risk of minor congenital malformations associated with use of prenatal prescription opioids in trimester 3, which seems to be dose-dependent. Further investigation is needed to establish causality and explore the physiologic plausibility of the association.

## Key Points

**Question:**

Is there an association between prenatal prescription opioid exposure and the risk of major and minor congenital malformations?

**Findings:**

In this cohort study of 12 424 pregnancies, 891 women were dispensed prescription opioids during pregnancy. Opioid exposure in trimester 1 was not associated with major birth defects, but women dispensed opioids in trimester 3 had higher risks of offspring with minor birth defects in the musculoskeletal system.

**Meaning:**

The study findings suggest a higher risk of minor congenital malformations in the musculoskeletal system that is associated with prenatal prescription opioid exposure in the third trimester; this risk appears to be dose-dependent.

## Teaser

This cohort study examines the incidence of major and minor congenital malformations in infants born to women who receive opioids for medical indications during pregnancy.

## Introduction

Pharmacologic analgesia plays a role in pain treatment during pregnancy.^1,2^ The American College of Obstetricians and Gynecologists recommends that alternative pain management strategies should be prioritized to avoid or minimize opioid exposure during gestation.^3^ However, common opioid use continues, presumedly because the benefits of treatment are considered to outweigh the risks of harm to both mother and infant.^4,5^

Studies are limited, and results are controversial regarding the effects of prenatal prescription opioid exposure on the risk of congenital malformations. Two recent large cohort studies reported that prenatal opioid exposure in the first trimester is not associated with a higher risk of major congenital malformations,^6,7^ but codeine use during early pregnancy was associated with a higher risk of spina bifida.^7^ Other studies suggested that gestational opioid exposure is associated with the risk of congenital malformations.^8,9,10^ Thus, whether opioid use in pregnancy is associated with major congenital malformations remains unclear. The goal of this study was to evaluate the risk of congenital malformations associated with gestational prescription opioid exposure among pregnant women enrolled in the Rhode Island state Medicaid program.

## Methods

### Data Sources

This retrospective cohort study was conducted with linked Rhode Island Medicaid claims and vital statistics data provided by the Rhode Island Department of Health and Rhode Island Executive Office of Health & Human Services. Details about the data linkage and data fields are described elsewhere.^11^ This study was designated exempt and informed consent was waived by the institutional review boards at the Rhode Island Department of Health and the University of Rhode Island because the study data were deidentified. This study followed the Strengthening the Reporting of Observational Studies in Epidemiology (STROBE) reporting guideline for cohort studies.

### Study Design and Cohort

We included mothers enrolled in Rhode Island Medicaid who had a live birth occurring between January 1, 2008, and December 31, 2016. Data analysis was conducted from May 1, 2019, to May 31, 2020. Eligible mothers were required to have continuous Rhode Island state Medicaid coverage from 3 months before the estimated conception date and throughout the pregnancy. Women were excluded if they had a cancer diagnosis or chromosomal abnormalities (*International Classification of Diseases, Ninth Revision, Clinical Modification* [*ICD-9-CM*] codes 758, 759.81, 759.82, and 759.83), had an opioid use disorder diagnosis, or received any medication-assisted treatments for opioid use disorder from 3 months before the conception date to the end of the pregnancy to avoid confounding from opioid use disorders. We excluded women exposed to the following teratogenic agents at any time during the baseline and pregnancy periods: isotretinoin, bexarotene, etretinate, phenobarbital, valproate, lithium, tazarotene, warfarin, misoprostol, mycophenolate, and thalidomide. To avoid exposure misclassification, we further excluded women dispensed prescription opioids during the 3-month baseline window, but not during the pregnancy.

### Assessment of Exposure

Prescription opioid exposure data were obtained through outpatient pharmacy claims reported by Rhode Island Medicaid using National Drug Codes, therapeutic class codes, or drug names. To separate outcomes of opioid use from major and minor congenital malformations, exposure was defined based on the etiologically relevant exposure window. Exposure was defined as having at least 1 day’s supply of a prescription opioid anytime during pregnancy trimesters 1, 2, or 3. Gestational age was derived through ultrasonographic examination of mothers and was a component of the Rhode Island Department of Health birth certificate data. Conception date was estimated by subtracting the infant’s gestational age, derived by ultrasonographic examination, from the infant’s date of birth. The first 3 months before the conception date were evaluated for baseline characteristics and use of medication. The pregnancy window included the period from the conception date through the delivery date. Trimester 1 was defined as from the conception date through 90 days after the conception date, trimester 2 was from 91 to 180 days after the conception date, and trimester 3 was from 181 days after the conception date to the delivery date. The comparison group included all mothers who had no prescription opioids dispensed during the baseline and pregnancy window.

### Outcome Definition

In this study, we assessed major and minor congenital malformations separately. Major congenital malformations included 47 birth defects that were defined by surveillance guidelines developed by the National Birth Defects Prevention Network.^12^ Cases of major congenital malformations were reported by Rhode Island birthing hospitals and ascertained by the Rhode Island Birth Defects Program through medical records review.^13^ All minor congenital malformations were identified using *ICD-9-CM* or *International Statistical Classification of Diseases, Tenth Revision, Clinical Modification* (*ICD-10-CM*) diagnosis codes from the infant’s Medicaid claims (eTable 1 in the Supplement) after excluding all major congenital malformations.^14,15^ Primary study outcomes included overall major or minor congenital malformations, defined as 1 or more major or minor congenital malformations. Secondary outcomes were defined as 10 specific categories of major or minor congenital malformations classified by organ system (eTable 1 in the Supplement).^16,17,18,19^ Classification was based on the *ICD-10-CM* codes and corresponding *ICD-9-CM* codes developed by the World Health Organization and adapted by the Centers for Disease Control and Prevention.^15,20^ This classification of birth defects has been implemented in many state birth defects monitoring systems and applied and validated in drug teratogenicity studies.^21,22,23,24,25^

Potential demographic and clinical risk factors were assessed 3 months before the conception date and controlled for in the analyses. Covariates taken into consideration were classified into several categories, including maternal demographic characteristics, substance use disorders, pain conditions, medical history, and concomitant drug use during baseline and the pregnancy window.^26,27^ Most covariates (eg, maternal tobacco use, alcohol consumption, other substance use, comorbidities, and comedication use) were identified using *ICD-9-CM* or *ICD-10-CM* codes, National Drug Codes, therapeutic class codes, or drug names from the Medicaid claims. Maternal age, year of delivery, and multiple births were extracted from the Rhode Island vital statistics data.

### Statistical Analysis

Descriptive analyses of baseline demographic and clinical variables were conducted. Continuous variables are presented as mean (SD) and compared using a 2-tailed, unpaired *t* test. Categorical variables are presented as frequency and percentage and compared using a χ^2^ or Fisher exact test.

A propensity score, fine-stratification approach with 50 strata was adopted to adjust for multiple confounding factors.^28,29^ Multivariable logistic regression analyses with propensity score fine-stratification weighting were applied to obtain the final estimates and corresponding 95% CIs. Adjusted relative risk (aRR) values were obtained using the log link function and binomial distribution.

Numerous prespecified sensitivity analyses were performed. First, dose-response relationships were examined for opioids dispensed with different cumulative opioid doses. A cumulative opioid dose for each exposed mother was calculated using morphine milligram equivalents (MMEs).^30^ A daily MME per prescription was obtained using a product of daily dose per prescription and the MME conversion factor for each opioid. The sum of the daily MME per prescription was determined in each pregnancy trimester to obtain a cumulative MME per day for every patient. Median cumulative MME per day was identified in each trimester and used as an empirical cutoff to dichotomize exposed mothers into 2 groups: high and low exposure.

Second, we examined associations between study outcomes and the 4 most commonly used opioid drugs: hydrocodone, oxycodone, codeine, and tramadol.^11^ Women with 2 or more opioids dispensed during pregnancy were excluded from this sensitivity analysis.

Third, to reveal the risk of individual prescription opioids associated with specific malformations and dose responses, we further categorized the study outcomes and identified the diagnosed indications that occurred with high incidence rates. We examined the association of these specific outcomes with individual opioid drugs.

All analyses were conducted using SAS, version 9.4 (SAS Inc). The level at which statistical significance was set was *P* ≤ .05.

## Results

Of 13 684 live births from January 1, 2008, to December 31, 2016, 10 768 mothers were enrolled in the Rhode Island Medicaid program. The final study cohort included 12 424 infants, consisting of 11 533 (92.8%) born to unexposed mothers and 891 (7.2%) born to mothers dispensed at least 1 prescription opioid during pregnancy (eFigure in the Supplement).

Of the exposed infants, 433 (48.6%) were exposed to prescription opioids in trimester 1, 306 (34.3%) in trimester 2, 306 (34.3%) in trimester 3, and 42 (4.7%) in all 3 trimesters. Before the propensity score method was applied (Table 1), exposed mothers in trimester 1, 2, or 3 were older; had higher prevalence of substance use, chronic diseases, mental disorders, and pain conditions; and were dispensed more concomitant medications at baseline and during pregnancy. After propensity score stratifying and weighting, balance was achieved among all confounding factors, indicated by the weighted standardized difference less than 10% between 2 comparison groups in the 3 trimesters (Table 1).

**Table 1.** Selected Demographic and Clinical Characteristics of the Study Population^a^

Characteristic	Unexposed [Reference] (n = 11 533)	Trimester 1			Trimester 2			Trimester 3		
		Exposed during trimester 1 (n = 433)	Standardized difference		Exposed during trimester 2 (N = 306)	Standardized difference		Exposed during trimester 3 (N = 306)	Standardized difference	
			Before PS adjustment	After PS adjustment		Before PS adjustment	After PS adjustment		Before PS adjustment	After PS adjustment
Age, mean (SD), y	25.7 (6.5)	28 (6.5)	0.37^b^	0.02	27 (6.2)	0.21^b^	−0.03	27.9 (5.9)	0.35^b^	0.02
Obesity, No. (%)	1104 (9.6)	65 (15.0)	0.17^b^	0.01	38 (12.4)	0.09	0.01	39 (12.7)	0.10	−0.01
Multiple births, No. (%)	298 (2.6)	18 (4.2)	0.09^b^	0.01	12 (3.9)	0.08	0	8 (2.6)	0	0
Tobacco use, No. (%)	1325 (11.5)	114 (26.3)	0.39^b^	0	76 (24.8)	0.35^b^	−0.02	85 (27.8)	0.42^b^	−0.01
Alcohol use, No. (%)	199 (1.7)	12 (2.8)	0.07^b^	−0.03	9 (2.9)	0.08	0	13 (4.2)	0.15^b^	−0.01
Other substance abuse, No. (%)^c^	200 (1.7)	22 (5.1)	0.19^b^	0.02	16 (5.2)	0.19^b^	0.01	20 (6.5)	0.24^b^	0.01
Lower back pain, No. (%)	2478 (21.5)	229 (52.9)	0.69^b^	−0.02	139 (45.4)	0.52^b^	0.03	132 (43.1)	0.48^b^	0
Headache, No. (%)	1927 (16.7)	134 (30.9)	0.34^b^	−0.02	85 (27.8)	0.27^b^	−0.02	94 (30.7)	0.33^b^	0
Chronic pelvic pain, No. (%)	1765 (15.3)	112 (25.9)	0.26^a^	−0.02	69 (22.5)	0.19^b^	−0.01	73 (23.9)	0.22^b^	−0.01
Fibromyalgia, No. (%)	204 (1.8)	37 (8.5)	0.31^b^	0	27 (8.8)	0.32^b^	0	23 (7.5)	0.28^b^	−0.02
Diabetes, No. (%)	295 (2.6)	19 (4.4)	0.10^b^	0	9 (2.9)	0.02	−0.01	11 (3.6)	0.06	−0.01
Hypertension, No. (%)	362 (3.1)	28 (6.5)	0.16^b^	−0.01	21 (6.9)	0.17^b^	−0.02	14 (4.6)	0.07	−0.01
Depression, No. (%)	2379 (20.6)	150 (34.6)	0.32^b^	−0.01	104 (34)	0.30^b^	−0.03	104 (34)	0.30^b^	−0.04
Anxiety, No. (%)	1906 (16.5)	141 (32.6)	0.38^b^	−0.02	93 (30.4)	0.33^b^	−0.01	91 (29.7)	0.32^b^	−0.03
Bipolar disorder, No. (%)	417 (3.6)	36 (8.3)	0.20^b^	0	15 (4.9)	0.06	−0.02	25 (8.2)	0.19^b^	0
ADHD, No. (%)	312 (2.7)	34 (7.9)	0.23^b^	0	18 (5.9)	0.16^b^	−0.01	16 (5.2)	0.13^b^	−0.02
Menstrual disorders, No. (%)	3295 (28.6)	164 (37.9)	0.20^b^	−0.01	113 (36.9)	0.18^b^	−0.02	106 (34.6)	0.13^b^	−0.03
Use in baseline or trimester 1, No. (%)										
Antidepressants	914 (7.9)	91 (21)	0.38^b^	−0.02	66 (21.6)	0.38^b^	−0.03	63 (20.6)	0.35^b^	−0.04
Antipsychotics	124 (1.1)	15 (3.5)	0.16^b^	−0.03	9 (2.9)	0.13^b^	0.01	10 (3.3)	0.15^b^	0.01
Benzodiazepines	459 (4)	72 (16.6)	0.43^b^	−0.01	49 (16)	0.41^b^	0	44 (14.4)	0.38^b^	−0.02
Anticonvulsants	206 (1.8)	44 (10.2)	0.36^b^	0	21 (6.9)	0.25^b^	−0.01	20 (6.5)	0.24^b^	0

Abbreviations: ADHD, attention-deficit/hyperactivity disorder; PS, propensity score.

^a^ Characteristics with fewer than 11 occurrences were not reported.

^b^ Statistically significant at *P* < .05.

^c^ Included cocaine or marijuana use.

Crude rates of outcome events and aRRs are presented in Table 2. Overall major birth defects occurred at a similar rate among exposed compared with unexposed women (aRR, 1.40; 95% CI, 0.84-2.34). When stratifying major birth defects by organ systems, similar event rates in comparison groups persisted. However, overall minor birth defects were significantly different in patients with opioid exposure in trimester 3 vs opioid nonexposure (aRR, 1.26; 95% CI, 1.04-1.53). After examining minor outcomes in 10 specific organ systems, a significantly increased risk of minor birth defects in the musculoskeletal system was observed in mothers with opioid exposure in trimester 2 (aRR, 1.50; 95% CI, 1.10-2.03) and trimester 3 (aRR, 1.65; 95% CI, 1.23-2.22).

**Table 2.** Crude Event Rates and Adjusted RR of Major and Minor Congenital Malformations^a^

Opioid exposure window	Outcome	Crude event rates, No. (%)		Adjusted RR (95% CI)^b^
		Unexposed	Exposed	
Trimester 1: unexposed (n = 11 533) vs exposed (n = 433)	Overall major birth defects	433 (3.8)	17 (3.9)	1.40 (0.84-2.34)
Trimester 2: unexposed (n = 11 533) vs exposed (n = 306)	Overall minor birth defects	2464 (21.4)	77 (25.2)	1.11 (0.91-1.37)
	Minor birth defects in the musculoskeletal system	984 (8.5)	40 (13.1)	1.50 (1.10-2.03)
Trimester 3: unexposed (n = 11 533) vs exposed (n = 306)	Overall minor birth defects	2464 (21.4)	85 (27.8)	1.26 (1.04-1.53)
	Minor birth defects in the musculoskeletal system	984 (8.5)	44 (14.4)	1.65 (1.23-2.22)

Abbreviation: RR, relative risk.

^a^ For each birth defect category, only significant results are presented.

^b^ Covariates adjusted for in the propensity score model included age; obesity; multiple births; tobacco use; alcohol use; other substance abuse; lower back pain; headache; chronic pelvic pain; fibromyalgia; history of diabetes, hypertension, depression, anxiety, bipolar disorder, attention-deficit/hyperactivity disorder, and menstrual disorders; and use of comedications during the baseline or exposure trimester, including antidepressants, antipsychotics, benzodiazepines, and anticonvulsants.

Table 3 presents the results of the dose-response effects examined using categorized cumulative MME stratified by high or low exposure by the median in each trimester. The median cumulative MME for trimester 1 was 30 mg/d; trimester 2, 37.5 mg/d; and trimester 3, 42.25 mg/d. Baseline characteristics comparing high or low exposure with no exposure in the 3 trimesters are presented in eTable 2 in the Supplement. High-dose opioid exposure and overall minor birth defects showed significant dose responses in trimester 3 (aRR, 1.34; 95% CI, 1.03-1.73). Similar patterns were observed for minor congenital anomalies of the musculoskeletal system, which were related to higher opioid doses in trimester 2 (aRR, 1.74; 95% CI, 1.20-2.53) and trimester 3 (aRR, 2.00; 95% CI, 1.38-2.91).

**Table 3.** Adjusted RR of Major or Minor Congenital Malformations Associated With High or Low MME Prescription Opioid Exposure^a^

Exposure window	Exposure	Outcome	Event rates, No. (%)		Adjusted RR (95% CI)^b^
			Unexposed	Exposed	
Trimester 1	Cumulative MME ≥30 (n = 252) vs unexposed (n = 11 533)	Overall major birth defects	433 (3.75)	11 (4.37)	1.53 (0.81-2.88)
	Cumulative MME <30 (n = 181) vs unexposed (n = 11 533)	Overall major birth defects	433 (3.75)	NA	1.09 (0.45-2.63)
Trimester 2	Cumulative MME ≥37.5 (n = 171) vs unexposed (n = 11 533)	Overall minor birth defects	2464 (21.36)	46 (26.90)	1.22 (0.94-1.57)
		Minor birth defects in musculoskeletal system	984 (8.53)	26 (15.20)	1.74 (1.20-2.53)
	Cumulative MME <37.5 (n = 135) vs unexposed (n = 11 533)	Overall minor birth defects	2464 (21.36)	31 (22.96)	1.02 (0.74-1.40)
		Minor birth defects in musculoskeletal system	984 (8.53)	14 (10.37)	1.16 (0.70-1.92)
Trimester 3	Cumulative MME ≥42.25 (n = 153) vs unexposed (n = 11 533)	Overall minor birth defects	2464 (21.36)	45 (29.41)	1.34 (1.03-1.73)
		Minor birth defects in musculoskeletal system	984 (8.53)	26 (16.99)	2.00 (1.38-2.91)
	Cumulative MME <42.25 (n = 153) vs unexposed (n = 11 533)	Overall minor birth defects	2464 (21.36)	40 (26.14)	1.18 (0.89-1.56)
		Minor birth defects in musculoskeletal system	984 (8.53)	18 (11.76)	1.36 (0.87-2.13)

Abbreviations: MME, morphine milligram equivalent; NA, not available; RR, relative risk.

^a^ The frequencies and percentages of outcome events are not presented if the numbers are lower than 10.

^b^ Covariates adjusted for in the propensity score model included age; obesity; multiple births; tobacco use; alcohol use; other substance abuse; lower back pain; headache; chronic pelvic pain; fibromyalgia; history of diabetes, hypertension, depression, anxiety, bipolar disorder, attention-deficit/hyperactivity disorder, and menstrual disorders; and use of comedications during the baseline or exposure trimester, including antidepressants, antipsychotics, benzodiazepines, and anticonvulsants.

Maternal exposure to hydrocodone or codeine in trimester 2 was linked to higher risks of minor birth defects in the musculoskeletal system (hydrocodone: aRR, 1.62; 95% CI, 1.01-2.59; codeine: aRR, 2.24; 95% CI, 1.27-3.05). In trimester 3, codeine use was associated with overall minor birth defects (aRR, 1.45; 95% CI, 1.04-2.00) and oxycodone use was associated with minor birth defects in the musculoskeletal system (aRR, 1.77; 95% CI, 1.14-2.76) (Table 4).

**Table 4.** Significant Adjusted RR of Major or Minor Congenital Malformations Associated With Exposure to Specific Opioids

Opioid drug	Outcome	Event rate, No. (%)		Adjusted RR (95% CI)^a^
		Unexposed	Exposed	
Trimester 2				
Hydrocodone	Minor birth defects in the musculoskeletal system	984 (8.53)	16 (13.22)	1.62 (1.01-2.59)
Codeine	Minor birth defects in the musculoskeletal system	984 (8.53)	11 (16.42)	2.24 (1.27-3.05)
Trimester 3				
Codeine	Overall minor birth defects	2464 (21.36)	26 (30)	1.45 (1.04-2.00)
Oxycodone	Minor birth defects in the musculoskeletal system	984 (8.53)	18 (15.38)	1.77 (1.14-2.76)

Abbreviation: RR, relative risk.

^a^ Covariates adjusted for in the propensity score model included age; obesity; multiple births; tobacco use; alcohol use; other substance abuse; lower back pain; headache; chronic pelvic pain; fibromyalgia; history of diabetes, hypertension, depression, anxiety, bipolar disorder, attention-deficit/hyperactivity disorder, and menstrual disorders; and use of comedications during the baseline or exposure trimester, including antidepressants, antipsychotics, benzodiazepines, and anticonvulsants.

Three minor anomalies in the musculoskeletal system that occurred in high frequencies and were associated with opioid exposure in trimester 2 or 3 were plagiocephaly, polydactyly, and other specified congenital deformities of the hip. Of those exposed to prescription opioids in trimester 2 or 3, a total of 45 infants were diagnosed with these 3 anomalies and 30 of these infants (66.7%) had plagiocephaly. Adjusted results showed that a higher risk of these musculoskeletal anomalies was linked to a high-dose maternal opioid exposure in trimesters 2 and 3. Hydrocodone use in trimester 2 (aRR, 3.01; 95% CI, 1.80-5.03) and oxycodone use in trimester 3 (aRR, 2.43; 95% CI, 1.37-4.02) were associated with these 3 anomalies (Table 5).

**Table 5.** Adjusted RR of Specific Minor Congenital Malformations in the Musculoskeletal System Associated With MME Dose and Individual Prescription Opioid Exposure^**a**^

Exposure window	Exposure	Event rate, No. (%)		Adjusted RR (95% CI)^b^
		Unexposed	Exposed	
MME dose				
Trimester 2	Cumulative MME≥37.5 (n = 171) vs unexposed (n = 11 533)	578 (5.01)	19 (11.11)	2.49 (1.58-3.94)
Trimester 3	Cumulative MME≥42.25 (n = 171) vs unexposed (n = 11 533)	578 (5.01)	16 (10.46)	2.56 (1.57-4.16)
Specific opioids				
Trimester 2	Hydrocodone (n = 121) vs unexposed (n = 11 533)	578 (5.01)	14 (11.57)	3.01 (1.80-5.03)
Trimester 3	Oxycodone (n = 117) vs unexposed (n = 11 533)	578 (5.01)	13 (11.11)	2.43 (1.37-4.02)

Abbreviations: MME, morphine milligram equivalent; RR, relative risk.

^a^ Specific minor congenital malformations in musculoskeletal system included plagiocephaly or congenital musculoskeletal deformities of the skull, face, and jaw; other specified congenital deformities of the hip; and polydactyly of fingers.

^b^ Covariates adjusted for in the propensity score model included age, obesity, multiple births, tobacco use, alcohol use, other substance abuse, lower back pain, headache, chronic pelvic pain, and fibromyalgia; history of diabetes, hypertension, depression, anxiety, bipolar disorder, attention-deficit/hyperactivity disorder, and menstrual disorder; and use of comedications during the baseline or exposure trimester, including antidepressants, antipsychotics, benzodiazepines, and anticonvulsants.

## Discussion

This study found that opioid dispensing in the first trimester was associated with no excess risk of major birth defects. Opioid exposure in the third trimester was associated with a 1.26-fold higher risk of overall minor congenital malformations compared with no exposure. After classifying by organ systems, prenatal opioid exposure in the second trimester was associated with a 1.50-fold higher risk and in the third trimester was associated with a 1.65-fold higher risk of minor malformations in the musculoskeletal system. The risk of minor birth defects overall or in the musculoskeletal system was higher with greater exposure in the third trimester. Such a dose-exposure effect would be expected of a teratogen, an agent that causes fetal malformation.

The prevalence of major congenital malformation is approximately 3% among live births in the US^31^; the reported incidence of minor congenital anomalies in newborns is more frequent and ranges from 14.7% to 47.7%, depending on population, defect types, and method of ascertainment.^32,33,34,35,36,37,38^ Infants with minor anomalies, mostly consisting of structural changes that pose less serious morbidity and disability, have a greater risk of more serious major malformations, developmental problems, or mental disorders.^32,34,39,40,41^ To our knowledge, no previous studies have examined the risk of minor anomalies in children with prenatal opioid exposure.

Consistent with teratology theory,^42^ our study suggests that minor congenital malformations in the musculoskeletal system are significantly associated with maternal opioid dispensing during the second or third trimester in a dose-dependent manner. Three anomalies that were disproportionately high in infants with prenatal opioid exposure in the second or third trimester were plagiocephaly, polydactyly, and other specified congenital deformities of the hip. The musculoskeletal system appears to be the third most common organ system (approximately 16%) involved in congenital malformations, preceded by the circulatory system (26%) and genitourinary system (21%).^43,44,45^ Infants with deformational plagiocephaly exhibited a higher rate of developmental delay and cognitive dysfunction.^46,47,48^ One study reported the high incidence of torticollis in infants with neonatal abstinence syndrome.^49^ Opioid use in pregnancy has been associated with dislocated hip and musculoskeletal defects and polydactyly.^5,50,51^ In our study, a high risk of plagiocephaly, along with hip deformities and polydactyly, was associated with higher doses of opioid use in the second and third trimesters.

Previous studies have reported an association between gestational opioid analgesia and major congenital malformations. Broussard et al^8^ reported that opioids were associated with various congenital heart malformations, spina bifida, and gastroschisis. Källén and colleagues^52,53^ found associations of severe congenital malformations, cardiovascular defects, and clubfoot with opioids, including tramadol. Other studies suggested that codeine is related to a higher risk of overall birth defects, spina bifida, and cardiovascular defects.^51,54,55,56^ A recent study by Bateman et al^6^ suggested no association between prenatal opioid exposure in the first trimester and major congenital malformations. In our study, major congenital malformations were not associated with prenatal opioid use in the first trimester. Hydrocodone, oxycodone, and codeine use in trimester 2 or 3 were related to a higher risk of malformations in the musculoskeletal system. Although these findings are based on a small number of outcome events, hydrocodone, oxycodone, tramadol, and codeine in general showed higher risks of congenital malformations and toxic effects in the fetus.

Mechanisms causing opioid teratogenicity are important but frequently unknown. Earlier animal studies suggested that endogenous opioid growth factor interacting with the ζ opioid receptor reduces DNA synthesis in the organs’ cells, thus affecting organ development in the fetus.^57,58^ Morphine-induced birth defects in the central nervous system could result from neuroblast apoptosis that relates to upregulated Bax to Bcl-2 ratio and caspase-3 activity in the early stage of fetal development.^59^ An animal study observed the developmental toxic effects of prenatal codeine use at a dose 11-fold higher than the maximum human therapeutic dose.^60^ More mechanism-oriented studies need to be conducted to examine the role of commonly prescribed opioids as growth regulators in organs and tissues and the association between gestational opioid exposure and congenital malformations.

The association between iatrogenic opioid exposure and birth defects, whether major or minor, increases the importance of the reduction of prescribed opioids during pregnancy. The American College of Obstetricians and Gynecologists specifically recommends that opioid analgesia should be used only in cases of severe pain.^3^ However, comprehensive educational initiatives are necessary to remind obstetricians, nurse midwives, and any other clinician who cares for women in pregnancy, including in an emergency setting, that nonopioid modalities for pain management should be first-line therapy during pregnancy. Only in the setting of a failed first-line nonopioid regimen and persistent pain does the benefit of maternal opioid exposure clearly outweigh the risk of potential fetal congenital malformation. Furthermore, it appears advisable to limit the dose of treatment when opioids are needed.

### Limitations

This study has several limitations. First, outpatient pharmacy claims data indicated that opioid medication was dispensed to the patient but may not reflect what was taken. Inpatient or illicit opioid use was not captured within these data. Prescription opioids may also be obtained from relatives, friends, and acquaintances. Prescription sharing for opioids ranges from 5% to 51.9%.^61^ Second, some uncertainty is present in estimating the ultrasonographically confirmed gestational age and the gestational weeks for opioid use in different trimesters. However, most of the first trimesters should be largely represented, thereby supporting the estimated association in the first trimester between opioid use and major malformations, but minor malformations could be associated with opioid use later in pregnancy. Third, only live births were included in this study; stillbirths, miscarriages, and pregnancy terminations were not examined, Furthermore, all minor birth defects were assessed using infants’ Medicaid claims; maternal Medicaid records were not used. Fourth, although many studies examining this association are available, a definitive conclusion that opioid exposure is responsible for congenital malformations is not possible because other studies, like ours, are associative in design. Unmeasured confounding factors, including race/ethnicity, lifestyle, severity of pain, environmental exposures, folic acid supplementation before or during pregnancy, and other comorbidities, could not be considered. Confounding by indication could be an issue for the study comparing exposed with unexposed pregnancies. However, we adjusted for many pain conditions in the analyses. Fifth, statistical power was insufficient for some analyses because of the rare frequency of exposure and outcomes. Thus we were not able to assess the association between a specific opioid or MME dose and a specific type of major birth defect. In addition, this study comprised mothers and infants of lower socioeconomic status insured by the state Medicaid program. This limited population may affect the generalizability of the study results.

## Conclusions

Our findings suggest that prenatal exposure to prescription opioids in the first trimester is not associated with major birth defects. In the second and third trimesters, it is associated with a higher risk of minor congenital malformations in a dose-dependent manner. Weighing risks and benefits before prescribing opioid drugs to pregnant women or women of childbearing age (in case of unintended pregnancy) could help reduce harm to the fetus. Further investigation in observational studies with larger study cohorts and basic science research with animal models are warranted to provide further support.

## Notes

Supplement. **eTable 1.** Operational Definitions of Congenital Malformation Outcomes **eFigure.** Flowchart of Study Cohort Selection **eTable 2.** Standardized Differences of Patient Characteristics Comparing High or Low MME Group vs Unexposed in Three Trimesters After PS Adjustment
