# Supplementary material for: Association of Gestational Opioid Exposure and Risk of Major and Minor Congenital Malformations
Source: JAMA Netw Open. 2021 Apr 13;4(4):e215708. doi: 10.1001/jamanetworkopen.2021.5708 (PMC8044730; doi:10.1001/jamanetworkopen.2021.5708)
Supplement: Supplement. — eTable 1. Operational Definitions of Congenital Malformation Outcomes eFigure. Flowchart of Study Cohort Selection eTable 2. Standardized Differences of Patient Characteristics Comparing High or Low MME Group vs Unexposed in Three Trimesters After PS Adjustment [file jamanetwopen-e215708-s001.pdf]

Supplementary Online Content

Wen X, Belviso N, Murray E, Lewkowitz AK, Ward KE, Meador KJ. Association of gestational opioid exposure and risk of major and minor congenital malformations. *JAMA Netw Open*. 2021;4(4):e215708. doi:10.1001/jamanetworkopen.2021.5708

eTable 1. Operational Definitions of Congenital Malformation Outcomes

eFigure. Flow Chart of Study Cohort Selection

eTable 2. Standardized Differences of Patient Characteristics Comparing High or Low MME Group vs Unexposed in Three Trimesters After PS Adjustment

This supplementary material has been provided by the authors to give readers additional information about their work.

© 2021 Wen X et al. *JAMA Network Open.*

eTable 1. Operational Definitions of Congenital Malformation Outcomes.

Variables	Operational Definitions
Overall Major Birth Defects	≥1 major congenital malformations that were identified by RI DOH Birth Defects Program
Overall Minor Birth Defects	≥1 minor congenital malformations that were identified using ICD9/10 codes from Infant's Medicaid Claims and not collected by RI DOH Birth Defects Program
Major and Minor congenital malformations in specific organ systems	
Central Nervous System	740, 741, 742, Q00, Q01, Q02, Q03, Q04, Q05, Q06, Q07
Cleft Palate and Lip	749, Q35, Q36, Q37
Eye, Face, and Neck	743, 744, Q10, Q11, Q12, Q13, Q14, Q15, Q16, Q17, Q18
Gastrointestinal	750, 751, Q38, Q39, Q40, Q41, Q42, Q43, Q44, Q45
Genital Organs	752, Q50, Q51, Q52, Q53, Q54, Q55, Q56
Circulatory System	745, 746, 747, Q20, Q21, Q22, Q23, Q24, Q25, Q26, Q27, Q28
Musculoskeletal System	754, 755, 756, Q65, Q66, Q67, Q68, Q69, Q70-Q79
Respiratory System	748, Q30, Q31, Q32, Q33, Q34
Urinary System	753, Q60, Q61, Q62, Q63, Q64
Other Malformations*	757, 237.7, 759, Q80-Q89
Specific Outcomes that occurred in high rates in infants with prenatal opioid exposure	
Plagiocephaly or congenital musculoskeletal deformities of skull, face, and jaw	754.0, Q673
Other specified congenital deformities of hip	755.63, Q65.89
Polydactyly of fingers	755.01, Q69.0, Q69.1

*Note:

Other Malformations include congenital anomalies of the integument, neurofibromatosis**,** other and unspecified congenital anomalies, and fetus or newborn affected by maternal conditions.

© 2021 Wen X et al. *JAMA Network Open.*

eFigure 1. Flow Chart of Study Cohort Selection.

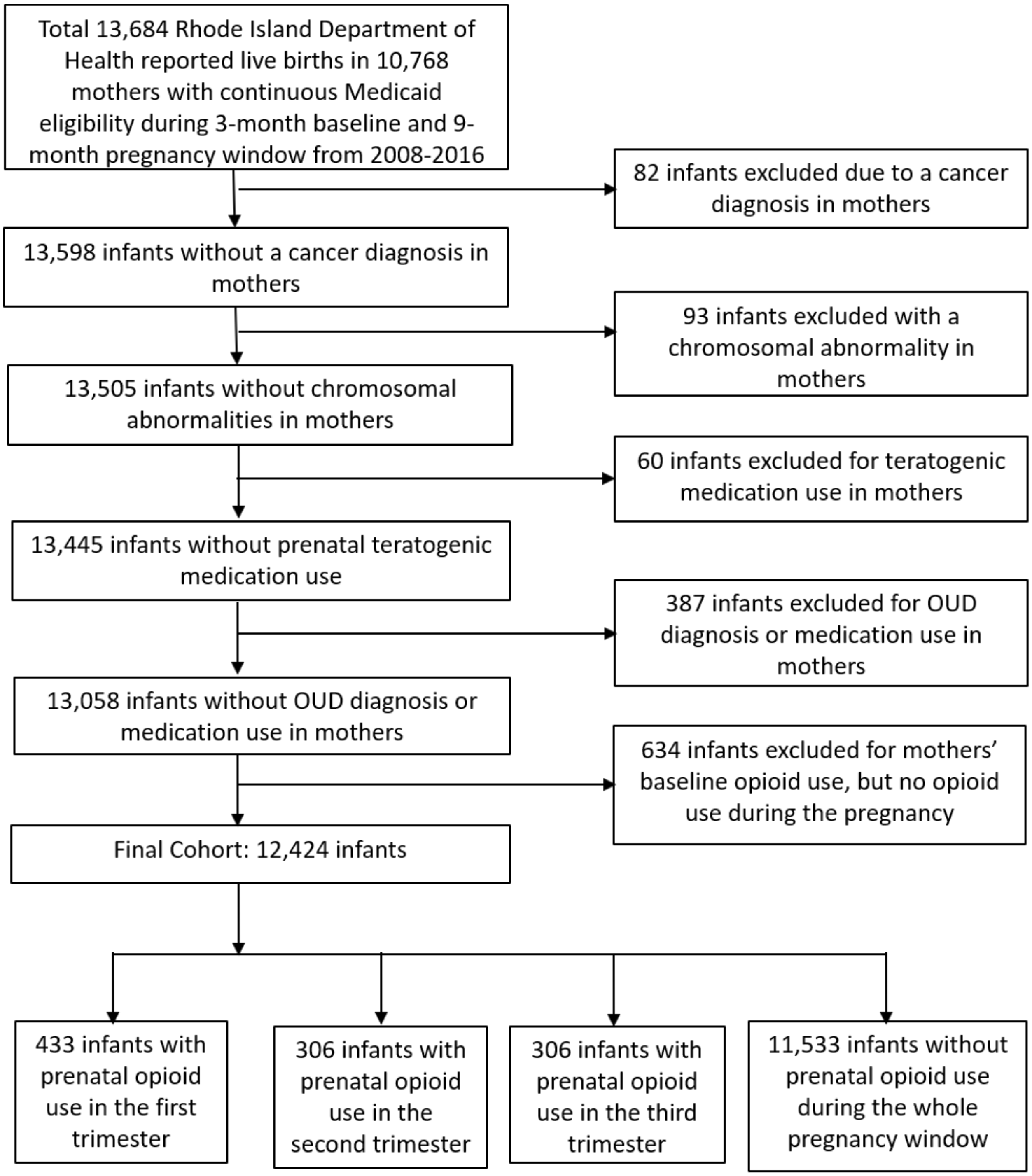

© 2021 Wen X et al. *JAMA Network Open.*

eTable 2. Standardized Differences of Patient Characteristics Comparing High or Low MME Group vs Unexposed in Three Trimesters after PS adjustment.

Characteristics	Trimester 1		Trimester 2		Trimester 3	
	Standardized Differences for High MME vs Unexposed	Standardized Differences for Low MME vs Unexposed	Standardized Differences for High MME vs Unexposed	Standardized Differences for Low MME vs Unexposed	Standardized Differences for High MME vs Unexposed	Standardized Differences for Low MME vs Unexposed
Age, Mean ± SD	.02	.05	-.01	-.06	.00	-.01
Obesity, N (%)	-.02	-.01	.00	.03	.02	-.02
Multiple Births, N (%)	-.01	.01	.00	-.01	.02	.00
Tobacco	.00	-.04	-.01	.02	-.01	-.03
Alcohol	.01	-.06	.00	.02	.04	.00
Other Substance Abuse	.03	.01	-.02	.00	.01	.00
Lower Back Pain	-.02	-.02	.01	.01	.02	-.01
Headache	-.02	-.02	.00	.00	.00	-.03
Chronic Pelvic Pain	.02	-.02	.00	.01	.03	-.01
Fibromyalgia	-.04	-.01	-.03	-.03	-.05	-.02
Diabetes	.01	.01	.00	.03	-.03	.00
Hypertension	.01	-.04	-.02	.00	.01	-.01
Depression	-.01	-.04	-.02	.00	.01	-.04
Anxiety	-.01	-.03	-.02	.02	-.03	.00
Bipolar	.02	.02	-.01	-.01	.00	-.01
ADHD	-.01	.03	.00	-.03	-.02	.03
Menstrual Disorders	-.01	.01	-.01	-.03	-.04	-.03
Antidepressants use in baseline or T1	-.01	-.03	-.04	.00	-.01	-.04
Antipsychotics use in baseline or Trimester 1	.01	.02	-.03	.03	.02	.04
Benzodiazepine use in baseline of Trimester 1	.01	-.01	-.01	-.01	-.02	-.01
Anticonvulsants use in baseline of Trimester 1	.01	.01	-.02	-.02	.00	-.01

© 2021 Wen X et al. *JAMA Network Open.*
